# Quantifying the health impacts of ambient air pollutants: recommendations of a WHO/Europe project

**DOI:** 10.1007/s00038-015-0690-y

**Published:** 2015-05-30

**Authors:** Marie-Eve Héroux, H. Ross Anderson, Richard Atkinson, Bert Brunekreef, Aaron Cohen, Francesco Forastiere, Fintan Hurley, Klea Katsouyanni, Daniel Krewski, Michal Krzyzanowski, Nino Künzli, Inga Mills, Xavier Querol, Bart Ostro, Heather Walton

**Affiliations:** WHO European Centre for Environment and Health, WHO Regional Office for Europe, Bonn, Germany; MRC-PHE Centre for Environment and Health, Population Health Research Institute, St George’s, University of London, London, UK; Institute for Risk Assessment Sciences, Universiteit Utrecht, Utrecht, The Netherlands; Julius Center for Primary Care and Health Sciences, University Medical Center Utrecht, Utrecht, The Netherlands; Health Effects Institute, Boston, USA; Dipartimento di Epidemiologia, ASL Roma E, Rome, Italy; Institute of Occupational Medicine, Riccarton, Edinburgh UK; Department of Hygiene, Epidemiology and Medical Statistics, University of Athens Medical School, Athens, Greece; McLaughlin Centre for Population Health Risk Assessment, University of Ottawa, Ottawa, ON Canada; Environmental Research Group, King’s College London, London, UK; Swiss Tropical and Public Health Institute, Basel, Switzerland; University of Basel, Basel, Switzerland; Centre for Radiation, Chemical and Environmental Hazards, Public Health England, Chilton, Didcot, Oxfordshire UK; Institute of Environmental Assessment and Water Research (IDÆA), Consejo Superior de Investigaciones Científicas (CSIC), Barcelona, Spain; Office of Environmental Health Hazard Assessment (OEHHA), Oakland, USA; MRC-PHE Centre for Environment and Health, and NIHR Biomedical Research Centre, Guy’s and St Thomas’ NHS Foundation Trust and King’s College London, London, UK

**Keywords:** Air pollutants, Health impact assessment, Cost–benefit analysis, Particulate matter, Ozone, Nitrogen dioxide

## Abstract

**Objective:**

Quantitative estimates of air pollution health impacts have become an increasingly critical input to policy decisions. The WHO project “Health risks of air pollution in Europe—HRAPIE” was implemented to provide the evidence-based concentration–response functions for quantifying air pollution health impacts to support the 2013 revision of the air quality policy for the European Union (EU).

**Methods:**

A group of experts convened by WHO Regional Office for Europe reviewed the accumulated primary research evidence together with some commissioned reviews and recommended concentration–response functions for air pollutant–health outcome pairs for which there was sufficient evidence for a causal association.

**Results:**

The concentration–response functions link several indicators of mortality and morbidity with short- and long-term exposure to particulate matter, ozone and nitrogen dioxide. The project also provides guidance on the use of these functions and associated baseline health information in the cost–benefit analysis.

**Conclusions:**

The project results provide the scientific basis for formulating policy actions to improve air quality and thereby reduce the burden of disease associated with air pollution in Europe.

**Electronic supplementary material:**

The online version of this article (doi:10.1007/s00038-015-0690-y) contains supplementary material, which is available to authorized users.

## Introduction

In 2013, WHO Regional Office for Europe coordinated two international projects: “Review of evidence on health aspects of air pollution—REVIHAAP” and “Health risks of air pollution in Europe—HRAPIE” in order to provide the European Commission (EC) and its stakeholders with evidence-based advice on the health aspects of air pollution for the review of the EU air quality policies. This advice was grounded in a review of the latest scientific evidence on the health effects of air pollutants and involved a large group of invited experts. The projects addressed a list of 26 key policy-relevant questions posed by the EC (WHO Regional office for Europe 2013a, b) and covering specific topics concerning health aspects of individual air pollutants and general aspects important to air quality management. Results of the REVIHAAP project provided an essential input to the HRAPIE project for which the task was to answer the following question: “What concentration–response functions for key pollutants should be included in cost–benefit analysis supporting the revision of EU air quality policy?”

Methods of air pollution health risk assessment have been well established in Europe since the first European project (Künzli et al. 2000) and adopted in many global projects including the first global burden of disease (GBD) study (Cohen et al. 2005; Lopez 2013) and the 2010 GBD update (Burnett et al. 2014; Lim et al. 2012). Concentration–response functions (CRFs) are necessary elements for the quantification of health impacts due to air pollution and require regular evaluation and update to incorporate new developments in science. In the specific case of the HRAPIE project, the CRFs were recommended for the quantification of impacts resulting from policies designed to reduce pollution concentrations. The recommended CRFs were subsequently used to identify the pollution reduction strategies that will most effectively deliver a benefit to health as part of the cost-effectiveness analysis (IIASA 2013). The impacts were monetized and then compared with the costs of interventions to form a cost–benefit analysis that enabled evidence-based policy making (Holland 2014). The HRAPIE project recommended the CRFs linking particulate matter (PM), ozone (O_3_) and nitrogen dioxide (NO_2_) concentrations with specific health effects and provided rationale for the decisions. In addition, the project indicated the background health data required for the quantification of effects. As well, the project recommended approaches to integrate specific health outcomes into the estimation of the total health burden of the exposure. The full results of the HRAPIE project are available in the project report (WHO Regional Office for Europe 2013b). The present paper provides a summary of the HRAPIE recommendations and a discussion on their implementation in the EC air policy package, which proposes a package of measures to reduce air pollution with a view of reducing impacts on human health and the environment in the EU.

## Methods

### Development process for HRAPIE project recommendations

We agreed to apply the established methods of air pollution health impact assessment as used and adapted in previous projects (Burnett et al. 2014; Cohen et al. 2005; Künzli et al. 2000; Lim et al. 2012). The focus of the methods was on the recommendation of CRFs. The discussions at WHO expert meetings provided general direction for the work on CRFs linking particulate matter with an aerodynamic diameter smaller than 2.5 µm (PM_2.5_) and O_3_ with mortality in cost-effectiveness analysis, as well as for the other outcomes to be considered for PM, O_3_ and NO_2_ in the cost–benefit analysis. Invited external reviewers selected to represent a range of countries and relevant disciplines also provided detailed comments on a draft version of the project report. The experts used the discussions from meetings and external reviewers’ comments to finalize the project conclusions.

Where available, recent systematic reviews or meta-analyses of epidemiological studies were used as input to the HRAPIE project (Hoek et al. 2012, 2013; Katsouyanni et al. 2009; Samoli et al. 2006; Weinmayr et al. 2010; WHO Regional Office for Europe 2013a). In some other cases, a dedicated meta-analysis was performed within the scope of the HRAPIE project, based on information collated by the Air Pollution Epidemiology Database (APED) at St. George’s, University of London (Anderson et al. 2007). This database contains details and results from all time-series studies of mortality and hospital admissions indexed in Medline, Embase, and Web of Knowledge until May 2011. Formal systematic reviews of the evidence were not available or feasible for all issues considered by HRAPIE, due to the limited resources of the project. Therefore, a few of the recommendations were formulated without a formal systematic review (such as for the effects of long-term PM10 exposure on postneonatal mortality and on incidence of chronic bronchitis in adults).

### Implementation of the HRAPIE project recommendations

The HRAPIE project provided recommendations for CRFs for which there is sufficient evidence for causality of associations according to the prior REVIHAAP project conclusions. It focussed on those air pollutants observed at concentrations causing health concerns, and which could be estimated at an appropriate spatial scale across Europe for the modelling of impacts. Each of the pollutant–outcome pairs recommended for cost–benefit analysis was classified into two categories:

- Group A: pollutant–outcome pairs for which enough data are available to enable quantification of effects;
- Group B: pollutant–outcome pairs for which there is more uncertainty about the data used for quantification of effects.

More information on the classification scheme is provided in the Online Resource. The recommendations considered specific conditions in EU countries—particularly in relation to the range of PM, O_3_ and NO_2_ concentrations expected to be observed in the EU in 2020—as well as the availability of baseline health data. Therefore, for the analysis in EU, no extrapolation beyond the range covered by epidemiological studies on the effects of ambient PM, O_3_ or NO_2_ was needed. However, generalization of the recommended approaches to other regions of the globe or individual countries, or to particular mixtures at the local level, may not be appropriate.

Meta-analyses were generally used to derive the coefficients. The random-effects model used in many meta-analyses makes no prior assumption of the homogeneity in distribution around the mean coefficient. The CIs associated with the recommended CRFs quantified the random error and the variability attributed to heterogeneity in the epidemiologic effect estimates used for health impact assessment; this is only a part of the total uncertainty in the risk estimates produced by the health impact assessment and cost–benefit analysis processes. Additional uncertainties were associated with other aspects of the overall process—for example, in the measurement and modelling of pollution, the estimates of background rates for morbidity or mortality and monetary valuation (WHO Regional Office for Europe 2013b; Holland 2014). Some less tangible issues arose from: (1) the transferability to the EU as a whole of CRFs and background rates from the locations where studies were carried out; (2) agreeing what particular pollutant–outcome pairs should be used together to estimate the health impacts of particular policies and measures; and (3) assessing the uncertainty of an overall estimate of effects, aggregated (after conversion to monetary values) over the various pollutant–outcome pairs.

An effort was made to give the best evidence-based estimate of the relationship between the pollutant and that health outcome for each pollutant–outcome pair included. However, some assumptions made by the experts might lead to underestimation of the effects. For example, acute effects of air pollution can occur with a delay (lag) of a few days to weeks but in selecting risk coefficients linking short-term exposures to health outcomes; however, the distributed lags were not taken into account, possibly resulting in underestimation of the overall effect. In addition, some health outcomes, such as low birth weight or lung function, for which there is evidence of air pollution effects were excluded because of difficulties in attaching monetary values. Further, the use of the limited set of CRFs proposed in Group A rather than the full set of CRFs may lead to underestimation of effect, as there is sufficient evidence of a causal relationship for pollutant–outcome pairs in both groups.

### Possible double counting of effects of various pollutants

Some of the effects of a pollutant on one health outcome may be captured in an estimate of effect on another health outcome or their group (e.g. mortality due to a specific cause is a part of all-cause mortality). To minimize double counting in the calculation of the total costs and benefits for a pollutant, we proposed a set of rules. We identified with an asterisk (*) those effect estimates that contributed to the total cost and benefits (and assumed that their effects are additive) in either the limited set (Group A*) or the extended set (Group B*) of effects. Those without the asterisk were considered to reflect the effects already accounted for by summing those with the asterisk.

Quantification of the health impacts of air pollution in the HRAPIE project focuses on three pollutants: PM (PM_2.5_, PM_10_) O_3_ and NO_2_. In the ambient air, those pollutants are usually correlated to some extent—sometimes negatively so, as with O_3_ and PM_10_ in winter (Katsouyanni et al. 2009). The REVIHAAP project therefore proposed quantification of health effects associated with these pollutants only after adjustment for at least one of the others (WHO Regional Office for Europe 2013a). Far fewer studies systematically applying two- or multi-pollutant modelling are available, however, than studies using single-pollutant models. Furthermore, effect estimates for a given pollutant derived from a multi-pollutant model may be subject to bias if the pollutants are correlated with each other and subject to measurement error (Fung and Krewski 1999). This can lead to underestimation of the RR for a pollutant of interest.

In balancing these considerations, the HRAPIE project generally opted for effect estimates based on the largest possible group of studies—i.e. reporting single-pollutant analyses—for quantification. Quantification of the impact for one pollutant from single-pollutant models may therefore include effects attributable to another with which it is correlated. Consequently, for any particular health outcome and exposure period (long-term or short-term exposure), estimated impacts of the three individual pollutants should not be added without recognizing that this will, in most practical circumstances, lead to some overestimation of the true impact. Impacts estimated for one pollutant only will, on the other hand, underestimate the true impact of the pollution mixture, if other pollutants affect that same health outcome independently.

## Results

The HRAPIE experts recommended CRFs linking long and short-term exposure to PM, O_3_ and NO_2_ with several indicators of mortality and morbidity (Table 1). Essential information concerning the CRFs, their sources and background health data necessary for estimation of health effects are provided in Table S1 in the Online Resource. Rationale for the selection of the CRFs and discussion of their uncertainties are presented in the full report of the HRAPIE project (WHO Regional Office for Europe 2013b).

**Table 1:** Pollutant–health outcome pairs for which HRAPIE project recommends concentration–response functions (modified from WHO 2013b)

Pollutant metric	Health outcome	Group	RR (95 % CI) per 10 µg/m^3^
PM_2.5_, annual mean	Mortality, all-cause (natural), age 30+ years	A*	1.062 (1.040–1.083)
PM_2.5_, annual mean	Mortality, cerebrovascular disease (includes stroke), ischaemic heart disease, COPD and trachea, bronchus and lung cancer, age 30+ years	A	GBD 2010 study (IHME 2013)^a^
PM_10_, annual mean	Postneonatal (age 1–12 months) infant mortality, all-cause	B*	1.04 (1.02, 1.07)
PM_10_, annual mean	Prevalence of bronchitis in children, age 6–12 (or 6–18) years	B*	1.08 (0.98–1.19)
PM_10_, annual mean	Incidence of chronic bronchitis in adults (age 18+ years)	B*	1.117 (1.040–1.189)
PM_2.5_, daily mean	Mortality, all-cause, all ages	A	1.0123 (1.0045–1.0201)
PM_2.5_, daily mean	Hospital admissions, CVDs (including stroke), all ages	A*	1.0091 (1.0017–1.0166)
PM_2.5_, daily mean	Hospital admissions, respiratory diseases, all ages	A*	1.0190 (0.9982–1.0402)
PM_2.5_, 2-week average, converted to PM_2.5_, annual average	RADs, all ages	B**	1.047 (1.042–1.053)
PM_2.5_, 2-week average, converted to PM_2.5_, annual average	Work days lost, working-age population (age 20–65 years)	B*	1.046 (1.039–1.053)
PM_10_, daily mean	Incidence of asthma symptoms in asthmatic children aged 5–19 years	B*	1.028 (1.006–1.051)
O_3_, summer months (April–September), average of daily maximum 8-h mean over 35 ppb	Mortality, respiratory diseases, age 30+ years	B	1.014 (1.005–1.024)
O_3_, daily maximum 8-h mean over 35 ppb	Mortality, all (natural) causes, all ages	A*	1.0029 (1.0014–1.0043)
O_3_, daily maximum 8-h mean over 10 ppb	Mortality, all (natural) causes, all ages	A	1.0029 (1.0014–1.0043)
O_3_, daily maximum 8-h mean over 35 ppb	Mortality, CVDs and respiratory diseases, all ages	A	CVD: 1.0049 (1.0013–1.0085); respiratory: 1.0029 (0.9989–1.0070)
O_3_, daily maximum 8-h mean over 10 ppb	Mortality, CVDs and respiratory diseases, all ages	A	CVD: 1.0049 (1.0013–1.0085); respiratory: 1.0029 (0.9989–1.0070)
O_3_, daily maximum 8-h mean over 35 ppb	Hospital admissions, CVDs (excluding stroke) and respiratory diseases, age 65+ years	A*	CVD: 1.0089 (1.0050–1.0127); respiratory: 1.0044 (1.0007–1.0083)
O_3_, daily maximum 8-h mean over 10 ppb	Hospital admissions, CVDs (excluding stroke) and respiratory diseases, age 65+ years	A	CVD: 1.0089 (1.0050–1.0127); respiratory: 1.0044 (1.0007–1.0083)
O_3_, daily maximum 8-h mean over 35 ppb	MRADs, all ages	B*	1.0154 (1.0060–1.0249)
O_3_, daily maximum 8-h mean over 10 ppb	MRADs, all ages	B	1.0154 (1.0060–1.0249)
NO_2_, annual mean over 20 µg/m^3^	Mortality, all (natural) causes, age 30+ years	B*	1.055 (1.031–1.080)
NO_2_, annual mean	Prevalence of bronchitic symptoms in asthmatic children aged 5–14 years	B*	1.021 (0.990–1.060) per 1 µg/m^3^ change in annual mean NO_2_
NO_2_, daily maximum 1-h mean	Mortality, all (natural) causes, all ages	A*	1.0027 (1.0016–1.0038)
NO_2_, daily maximum 1-h mean	Hospital admissions, respiratory diseases, all ages	A	1.0015 (0.9992–1.0038)
NO_2_, 24-h mean	Hospital admissions, respiratory diseases, all ages	A*	1.0180 (1.0115–1.0245)

*CI* confidence interval, *COPD* chronic obstructive pulmonary disease, *CVD* cardiovascular disease, *GBD* global burden of disease, *HRAPIE* health risks of air pollution in Europe, *MRAD* minor restricted activity days, *NO*_*2*_ nitrogen dioxide, *O*_*3*_ ozone, *PM*_*2.5*_ particulate matter with an aerodynamic diameter smaller than 2.5 µm, *PM*_*10*_ particulate matter with an aerodynamic diameter smaller than 10 µm, *ppb* parts per billion, *RAD* restricted activity days, *RR* relative risk

Group A: pollutant–outcome pairs contributing to the limited set of effects but considered already accounted for by summing those with an asterisk

Group A*: pollutant–outcome pairs contributing to the total limited set of effects (the effects are additive)

Group B: pollutant–outcome pairs contributing to the extended set of effects but considered already accounted for by summing those with an asterisk

Group B*: pollutant–outcome pairs contributing to the total extended set of effects (the effects are additive)

Group B**: Only residual RADs to be added to total effect, after days in hospital, work days lost and days with symptoms are accounted for

^a^Supra-linear exponential decay saturation model (age-specific), linearized by the PM_2.5_ expected in 2020 under the current legislation scenario

## Discussion

Since the HRAPIE project report was published, a number of policy-relevant questions have come to our attention, raised by readers of the report. The following paragraphs provide some further perspectives on the HRAPIE recommendations, in response to the issues raised.

### Use of thresholds, lower levels, counterfactuals, etc., in quantifying health impacts

The HRAPIE report recommended that quantification of long-term effects of PM_2.5_ on mortality be undertaken at all concentrations, whereas for NO_2_, quantification was recommended only above annual mean concentrations >20 µg/m^3^. This has raised some confusion.

We point out that following the recommendation for estimating impacts of PM_2.5_ down to zero concentrations, higher impacts are estimated than in the GBD 2010 study (Lim et al. 2012). This is because the GBD 2010 study estimated impacts down to a counterfactual concentration below which—because of a lack of data—the risk could not be quantified; this counterfactual for ambient air pollution is different than zero and was chosen as an alternative exposure distribution to which the current exposure distribution is compared (Burnett et al. 2014). The selection of counterfactual levels for risk factors, including for ambient particulate air pollution, were informed by: (1) a counterfactual population distribution of exposure that is theoretically possible and would result in the lowest population disease burden—the theoretical minimum risk exposure distribution (TMRED), and (2) the availability of convincing evidence supporting a continuous reduction in risk of disease extending from current levels down to the counterfactual TMRED (Murray et al. 2012). On this basis, the TMRED for ambient PM_2.5_ was defined by a uniform distribution with lower and upper bounds at the minimum and 5th percentile of the PM_2.5_ exposure distribution of the American Cancer Society (ACS) Cancer Prevention II cohort study of 5.8 and 8.8 µg/m^3^, respectively (Krewski et al. 2009; Lim et al. 2012; Burnett et al. 2014). This approach assumed that long-term exposure to PM_2.5_ less than 5.8 µg/m^3^ confers no excess risk, and that although excess risk may extend below the 5th percentile of the distribution, estimates in that range are statistically unstable and therefore highly uncertain. Uncertainty from the counterfactual, the prevalence of exposure, and the exposure–response function was all propagated into the final risk factor uncertainty, and the final uncertainty reflected that of the estimated age- and sex-specific mortality rates as well. For future work under GBD 2013 the same approach is used but includes information from 9 cohort studies with minimum exposure concentrations less than or equal to the 5th percentile in the ACS, i.e. 8.8 µg/m^3^. Whenever impacts of policy measures are estimated using the HRAPIE CRFs, results will not differ greatly from those obtained using the GBD approach, as for the medium to long term, concentrations in most, if not all, areas in Europe will not likely go below 5.8 µg/m^3^. We also point out that under the HRAPIE project, due to wider availability of risk estimates and greater precision of background national data for all-cause mortality in Europe, natural all-cause mortality was chosen as the outcome for quantification. This is different to what was chosen as outcome as part of the GBD project, where cause-specific mortality was used because patterns of causes of death vary considerably globally.

The lower limit of 20 µg/m^3^ (annual) for NO_2_ was motivated by reference to the Naess et al. (2007) Norwegian study, and by unpublished analyses of the data presented in the Cesaroni et al. (2013) paper from Rome. These studies assessed NO_2_ effects in single-pollutant models. The Naess et al. (2007) paper shows a generally linear relationship between NO_2_ and mortality, among 71–90 year olds, in the 20–60 µg/m^3^ range. Figure 1 in that paper actually shows a steeper CRF in the 0–20 µg/m^3^ range, with wider confidence intervals due to the smaller numbers of participants at such low exposures. The Cesaroni et al. (2013) paper continued to show a linear decline below 20 µg/m^3^, although again with wider confidence intervals. The evidence presented does not suggest that the effect is zero at 20 µg/m^3^, just that the size of the effect is less certain below 20 µg/m^3^. We also note that a recent cohort study conducted among ca. 52,000 adults in Copenhagen, Denmark (Raaschou-Nielsen et al. 2012) has shown a significant, almost linear concentration–response relationship between long-term NO_2_ concentration (chosen by the authors as an indicator of urban air pollution dominated by traffic exhaust) and mortality [for cardiovascular disease (CVD), ischemic heart disease and all causes] throughout the observed range of NO_2_ concentrations, which in the large majority of subjects was below 20 µg/m^3^ (minimum 10.5 µg/m^3^, median 15.1 µg/m^3^, maximum 59.6 µg/m^3^). This study was included in the Hoek et al. (2013) meta-analysis, but we did not explicitly consider it when discussing lower limits of quantification in the HRAPIE project. All-cause mortality increased by 8 % per 10 µg/m^3^ NO_2_ long-term exposure at the residence address in the study by Raaschou-Nielsen et al. (2012), so slightly more than estimated in the Hoek et al. meta-analysis. Therefore, the HRAPIE recommendation to calculate the impacts of long-term NO_2_ exposure on mortality for levels over 20 µg/m^3^, ignoring potential impacts at lower concentrations, may be too conservative.

### Age dependency of CRFs

The HRAPIE report briefly discussed reasons why RRs for factors such as smoking and CVD decline with age, and how that might impact calculations. Air pollution studies have not generally investigated age dependency of RRs in any detail, but there is some specific information to suggest that this is also true for relationships between air pollution and all-cause and cause-specific mortality. Over a 10-year follow-up period, an analysis of the ACS-1 study (Enstrom 2005) showed a RR for PM_2.5_ and all-cause mortality twice as high among subjects aged 43–64 years at baseline than in subjects aged 65–99 years at baseline (Table 6 of the paper). The study by Naess et al. (2007) showed, for both men and women, a clearly higher RR of CVD as well as chronic obstructive pulmonary disease (COPD) mortality in subjects aged 51–70 years at baseline than in subjects aged 71–90 years at baseline (Tables 3 and 4 of the paper). In the Harvard Six Cities Study, as reanalysed by Krewski et al. (2000), the RR for all-cause mortality in relation to an 18.6 µg/m^3^ increase in PM_2.5_ was 2.11 (0.88–5.07) for those <40 years old at baseline, 1.66 (1.17–2.35) for those 41–55 years old, and 1.17 (0.98–1.40) for those >55 years old at baseline. The same reanalysis showed that, in the ACS study, for those with high school education or less, the RR for all-cause mortality in relation to a 24.5 µg/m^3^ increase in PM_2.5_ was 1.51 (1.00–2.27) for those <50 years old at baseline, 1.27 (1.02–1.60) for those 50–60 years old, and 1.28 (1.14–1.43) for those >60 years old at baseline. Early work had already shown that impacts on life expectancy may be over-estimated when RRs observed at certain specific ages in cohort studies are applied to subjects at higher ages with high baseline mortality (Brunekreef, 1997). Most current risk assessments assume that the excess relative risk among adults does not vary with age, but the GBD 2010 estimates incorporated age-dependency of the air pollution relative risk(s) such that the age-specific excess relative risk for cardiovascular mortality declined with increasing age leading to lower estimates of attributable burden (Lim et al. 2012; Burnett et al. 2014).

### Cessation lag between reduced long-term exposure to PM_2.5_ and mortality

The distribution of the cessation lag is relevant both for the size of the effect over a defined time period and when calculating the economic value of the effect due to discounting the value over time. The HRAPIE report stated that findings from the follow-up of the Harvard Six Cities study suggest that mortality effects may be partially reversible, over a time period possibly as short as a year. Ideally, for health impact calculations, a range of possible delays between reduced exposure and a reduced impact on mortality would be used. The HRAPIE report noted that long-term exposure to PM_2.5_ is linked with lung cancer mortality. Delays for this are likely to be measured in decades, although lung cancer mortality is a small proportion of the total. Other organizations such as the United States Environment Protection Agency (US EPA) have recommended a distribution of different lags (US EPA 2011). Readers are referred to this and other documents (Hurley et al. 2005; COMEAP 2010) for a more detailed discussion.

### Application of HRAPIE recommendations and implications for European Union air policy

An impact assessment accompanying the EU policy package was developed by the EC and provides the results of the implementation of the HRAPIE recommendations in a cost–benefit context (EC 2013). According to the EC’s impact assessment, over 406,000 premature deaths were estimated to be related to long-term PM_2.5_ and short-term O_3_ exposure in year 2010. New evidence on impacts from chronic O_3_ exposure is not included but would add around 5 % to this total (EC 2013). The evidence of health impacts from NO_2_ exposure was considered, but as there was a lack of agreement regarding the extent to which the exposure data used by the EC properly reflected exposure of the population (Holland 2014), no quantification was made of NO_2_ health impacts. Further work is needed to characterize the link between estimated NO_2_ exposure and health outcomes as provided in the recommendations of the HRAPIE report.

According to the EC assessment, the mortality associated with PM, the most important pollutant, has been reduced by around 20 % between 2000 and 2010 (EC 2013). Modelled trends in pollutant levels show that under a business-as-usual scenario (baseline projection) the impacts of air pollution will continue to decrease by 2020, where they will amount to an estimated 340,000 premature deaths. The progress in further reducing the health impacts from air pollution is expected to be considerably slower beyond 2020. On average across the EU, baseline projection suggests a decline of the loss of statistical life expectancy attributable to the exposure to PM_2.5_ from 8.5 months in 2005 to 5.3 months in 2025. Depending on the valuation methodology [Value of Statistical Life (VSL) or Value of a Life-Year (VOLY)], the health-related external costs from air pollution ranged between €330 billion and €940 billion in 2010, and would be reduced in the baseline to €210–730 billion in 2030 (2005 € prices).

The corresponding benefits of the proposed air policy package can be monetized, resulting in about €40–140 billion per year in 2030, while the costs of pollution abatement to implement the package are estimated to reach € 3.4 billion per year in 2030. The impact assessment states that the monetized benefits will therefore be about 12–40 times higher than the costs (EC 2013).

The policy package does not propose changes to the existing air quality standards in the ambient air quality directive at this stage. The EC acknowledges that the current standards are insufficient for the protection of public health, particularly in reference to the WHO air quality guidelines. The focus will be on a full attainment of current air quality standards by 2020. The new policy proposes stricter national emission ceilings and new source legislations which, according to the EC, are expected to pave the way for tightened standards in the ambient air quality directive at a later stage. A 5-year policy review cycle is being considered with a first review taking place no later than 2020, at which time the scope for tightening the air quality standards will be considered by the EC (EC 2013).

### Conclusion

The scientific evidence is rapidly expanding, reaffirming and strengthening previously reported associations as well as revealing new health outcomes. Special efforts are required from the scientific community and the policy-makers to engage in a dialogue and enable proper interpretation and synthesis of the scientific evidence for use in policy formulation. The HRAPIE project report, summarized in this paper, illustrates the complexities involved in recommending suitable CRFs, with accompanying recommendations on methods, baseline rates and appropriate strategies for combining results. These complexities need to be acknowledged, and sufficient resources should be made available to enable proper synthesis and interpretation of the evidence in future reviews of air pollution health effects.

The REVIHAAP and HRAPIE projects provide scientific arguments for taking decisive actions to improve air quality to further reduce the burden of disease associated with air pollution in Europe. They further recommend that the EC ensures that the evidence on the health effects of air pollutants and the implications for its air quality policy is reviewed regularly. The material developed as part of these projects is equally relevant to all Member States of the EU, in their development and implementation of effective strategies to reduce air pollution and its significant impacts on public health. The two projects described above also provide an important input to the development of air quality policies by the parties of the United Nations Economic Commission for Europe (UNECE) Convention on Long-range Transboundary Air Pollution (LRTAP), which are outside the EU, especially Member States from the eastern part of the WHO European Region.

## Electronic supplementary material

Supplementary material 1 (DOCX 53 kb)
