# Supplementary material for: Quantifying the health impacts of ambient air pollutants: recommendations of a WHO/Europe project
Source: Int J Public Health. 2015 May 30;60(5):619–27. doi: 10.1007/s00038-015-0690-y (PMC4480843; doi:10.1007/s00038-015-0690-y)
Supplement: Supplementary file 1 — Supplementary material 1 (DOCX 53 kb) [file 38_2015_690_MOESM1_ESM.docx]

International Journal of Public Health

Online Resource

Quantifying the health impacts of ambient air pollutants: Recommendations of a WHO/Europe project

Marie-Eve Héroux, Hugh Ross Anderson, Richard Atkinson, Bert Brunekreef, Aaron Cohen, Francesco Forastiere, Fintan Hurley, Klea Katsouyanni, Daniel Krewski, Michal Krzyzanowski, Nino Künzli, Inga Mills, Xavier Querol, Bart Ostro, Heather Walton

Corresponding author: Marie-Eve Héroux, WHO European Centre for Environment and Health, WHO Regional Office for Europe, Bonn, Germany, Tel.: +49 228 815 0437, Fax: +49 228 815 0440, [herouxm@ecehbonn.euro.who.int](mailto:herouxm@ecehbonn.euro.who.int)

More information on the scheme developed in the Health risks of air pollution in Europe (HRAPIE) project for classification of the pollutant-outcome pairs recommended for cost-benefit analysis is provided in this Online Resource. Among the effect estimates (ESs) for pollutant–outcome pairs listed in Table S1, those marked with an asterisk (*) contribute to the total effect (i.e. the effects are additive) of either the limited set (Group A*) or the extended set (Group B*) of effects. The HRAPIE project recommended that the calculation of the range of overall costs and benefits be based on the following principles (WHO Regional Office for Europe, 2013b):

- the calculation of a limited set of impacts based on the sum (Σ) of Group A*;
- the range of uncertainty around the limited estimate, from Σ minimum (Group A*, Group A) to Σ maximum (Group A*, Group A), possibly combined with Monte Carlo estimates based on confidence intervals (CIs) of relative risks (RRs) – minimum/maximum functions select smaller/larger effect in the related alternative options;
- the calculation of an extended set of impacts based on Σ Group A* + Σ Group B*;
- the range of uncertainty around the extended estimate, from Σ [minimum (Group A*, Group A) + minimum (Group B*, Group B)] to Σ [maximum (Group A*, Group A) + maximum (Group B*, Group B)], possibly combined with Monte Carlo estimates based on CIs of RRs.

Table S1. Concentration-response functions (CRFs) recommended by the Health risks of air pollution in Europe (HRAPIE) project (ACS: American Cancer Society,

APHEA-2: Air Pollution and Health: a European Approach project, APHENA: Air Pollution and Health: a European and North American approach study, APED: Air Pollution Epidemiology Database, ASHMOG: Loma Linda University Adventist Health and Smog, CI: confidence interval, COPD: chronic obstructive pulmonary disease, CRF: concentration-response function, CVD: cardiovascular disease, GBD: Global Burden of Disease, ICD-9: International Classification of Diseases, ninth revision, ICD-10: International Classification of Diseases, tenth revision, ISAAC: International Study on Asthma and Allergies in Childhood, MDB: European mortality database, MRAD: minor restricted activity day, NO_2_: nitrogen dioxide, O_3_: ozone, OC: organic carbon, PATY: Pollution and the Young, PM: particulate matter, PM_2.5_: particulate matter with an aerodynamic diameter smaller than 2.5 µm, PM_10_: particulate matter with an aerodynamic diameter smaller than 10 µm, RAD: restricted activity day, RR: relative risk, SAPALDIA: Swiss Study on Air Pollution and Lung Disease in Adults, SD: standard deviation)

| PM, long-term exposure | | | | | | | |
| --- | --- | --- | --- | --- | --- | --- | --- |
| Pollutant metric | Health outcome | Group | R) (95% CI) per 10 µg/m^3^ | Range of pollutant concentration to be quantified | Source of background health data | Source of CRF | Comments |
| PM_2.5_, annual mean | Mortality, all-cause (natural), age 30+ years | A* | 1.062  (1.040–1.083) | All | MDB (WHO, 2013c), rates for deaths from all natural causes (ICD-10) chapters I–XVIII, codes A–R) in each of the 53 countries of the WHO European Region, latest available data | Meta-analysis of 13 cohort studies with results: Hoek et al. (2013) Coefficient from single-pollutant model |  |
| PM_2.5_, annual mean | Mortality, cerebrovascular disease (includes stroke), ischaemic heart disease, COPD and trachea, bronchus and lung cancer, age 30+ years | A | GBD 2010 study (IHME, 2013), supra-linear exponential decay saturation model (age-specific), linearized by the PM_2.5_ expected in 2020 under the current legislation scenario | All | European detailed mortality database (WHO, 2013d), ICD-10 codes cerebrovascular: I60–I63, I65–I67, I69.0–I69.3; ischaemic heart disease: I20–I25; COPD: J40–J44, J47; trachea, bronchus and lung cancer: C33–C34, D02.1–D02.2, D38.1 | CRFs used in the GBD 2010 study Coefficient from single-pollutant model | An alternative to all-cause mortality  Both age-specific and all-age estimates to be calculated to assess the potential effect of age stratification  Supra-linear exponential decay saturation model (age-specific), linearized by the PM_2.5_ expected in 2020 under the current legislation scenario |
| PM_10_, annual mean | Postneonatal (age 1–12 months) infant mortality, all-cause | B* | 1.04  (1.02, 1.07) | All | European Health for All database (WHO, 2013e) and United Nations projections | Woodruff, Grillo and Schoendorf (1997), based on 4 million infants in the United States  Coefficient from single-pollutant model | More recent analysis (Woodruff, Darrow and Parker, 2008) based on 3.5 million infants in the United States gives RR = 1.18 (1.06, 1.31) for respiratory postneonatal infant mortality; the older analysis is recommended as a source of RR due to unavailability of cause-specific postneonatal mortality data |
| PM_10_, annual mean | Prevalence of bronchitis in children, age 6–12 (or 6–18) years | B* | 1.08  (0.98–1.19) | All | Mean prevalence from the PATY study: 18.6% (range 6–41%) | PATY study (Hoek et al., 2012) analysing data from about 40 000 children living in nine countries Coefficient from single-pollutant model | Heterogeneity of the association (p<0.10) between studies |
| PM_10_, annual mean | Incidence of chronic bronchitis in adults (age 18+ years) | B* | 1.117  (1.040–1.189) | All | Annual incidence 3.9 per 1000 adults based on SAPALDIA | Combination of results from longitudinal studies AHSMOG and SAPALDIA Coefficient from single-pollutant model | Two studies with different odds ratios/RRs; cost–benefit analysis based on symptoms reporting is weak indication of clinically recognized COPD |

| PM, short-term exposure | | | | | | | |
| --- | --- | --- | --- | --- | --- | --- | --- |
| Pollutant metric | Health outcome | Group | RR (95% CI) per 10 µg/m^3^ | Range of pollutant concentration to be quantified | Source of background health data | Source of CRF | Comments |
| PM_2.5_, daily mean | Mortality, all-cause, all ages | A | 1.0123  (1.0045–1.0201) | All | MDB (WHO, 2013c) | APED meta-analysis of 12 single-city and one multicity studies  Coefficient from single-pollutant model | For information only: not proposed as an alternative to long-term PM_2.5_ exposure  The premature deaths attributed to short-term changes of PM_2.5_ are already accounted for in estimating the effects of long-term exposure |
| PM_2.5_, daily mean | Hospital admissions, CVDs (includes stroke), all ages | A* | 1.0091  (1.0017–1.0166) | All | European hospital morbidity database (WHO, 2013f), ICD, ninth revision (ICD-9) codes 390-459; ICD-10 codes I00–I99 | APED meta-analysis of four single-city and one multicity studies Coefficient from single-pollutant model |  |
| PM_2.5_, daily mean | Hospital admissions, respiratory diseases, all ages | A* | 1.0190  (0.9982–1.0402) | All | European hospital morbidity database (WHO, 2013f),  ICD-9 codes 460-519; ICD-10 codes J00–J99 | APED meta-analysis of three single-city studies Coefficient from single-pollutant model |  |
| PM_2.5_, two-week average, converted to PM_2.5_, annual average | RADs, all ages | B** | 1.047  (1.042–1.053) | All | 19 RADs per person per year: baseline rate from the Ostro and Rothschild (1989) study | Study of 12 000 adults followed for six years in 49 metropolitan areas of the United States (Ostro, 1987)  Coefficient from single-pollutant model | One 1987 study from the United States; no data of background rate in Europe |
| PM_2.5_, two-week average, converted to PM_2.5_, annual average | Work days lost, working-age population (age 20–65 years) | B* | 1.046  (1.039–1.053) | All | European Health for All database (WHO, 2013e) | Study of 12 000 adults followed for six years in 49 metropolitan areas of the United States (Ostro, 1987)  Coefficient from single-pollutant model | High variability of background rates based on reported sick absenteeism in Europe, reflecting intercountry differences in definition |
| PM_10_, daily mean | Incidence of asthma symptoms in asthmatic children aged 5–19 years | B* | 1.028  (1.006–1.051) | All | Prevalence of asthma in children based on “severe asthma” in ISAAC (Lai et al., 2009) – western Europe: 4.9%; northern and eastern Europe: 3.5%. Daily incidence of symptoms in this group: 17% (interpolation from several panel studies) | Meta-analysis of 36 panel studies of asthmatic children conducted in 51 populations, including 36 from Europe, (Weinmayr et al., 2010)  Coefficient from single-pollutant model | Varying definition of the target population and of the daily occurrence of symptoms |

** Only residual RADs to be added to total effect, after days in hospital, work days lost and days with symptoms are accounted for.

| O_3_, long-term exposure | | | | | | | |
| --- | --- | --- | --- | --- | --- | --- | --- |
| Pollutant metric | Health outcome | Group | RR (95% CI) per 10 µg/m^3^ | Range of pollutant concentration to be quantified | Source of background health data | Source of CRF | Comments |
| O_3_, summer months (April–September), average of daily maximum 8-hour mean over 35 parts per billion (ppb) | Mortality, respiratory diseases, age 30+ years | B | 1.014  (1.005–1.024) | >35 ppb (>70 µg/m³) | MDB (WHO, 2013c), ICD-10 codes J00–J99 | Single-pollutant models from ACS data analysis (Jerrett et al., 2009) | Alternative to effects of short-term O_3_ on all-cause mortality  Coefficient from single-pollutant model |

| O_3_, short-term exposure | | | | | | | | | | |
| --- | --- | --- | --- | --- | --- | --- | --- | --- | --- | --- |
| Pollutant metric | | Health outcome | | Group | RR (95% CI) per 10 µg/m^3^ | Range of pollutant concentration to be quantified | Source of background health data | | Source of CRF | Comments |
| O_3_, daily maximum 8-hour mean | | Mortality, all (natural) causes, all ages | | A* | 1.0029  (1.0014–1.0043) | >35 ppb (>70 µg/m³) | MDB (WHO, 2013c), ICD-10 chapters I–XVIII, codes A–R | | APHENA study, based on data from 32 European cities; coefficients adjusted for PM_10_ in two-pollutant model | APHENA study, based on full range of observed O_3_ concentrations, including levels <35 ppb; thus effects at O_3_ <35 ppb are ignored |
| O_3_, daily maximum 8-hour mean | | Mortality, all (natural) causes, all ages | | A | 1.0029  (1.0014–1.0043) | >10 ppb (>20 µg/m³) | MDB (WHO, 2013c), ICD-10 chapters I–XVIII, codes A–R | | APHENA study based on data from 32 European cities; coefficients adjusted for PM_10_ in two-pollutant model | Alternative to the assessment for O_3_ >35 ppb only |
| O_3_, daily maximum 8-hour mean | | Mortality, CVDs and respiratory diseases, all ages | | A | CVD: 1.0049 (1.0013–1.0085); respiratory: 1.0029  (0.9989–1.0070) | >35 ppb (>70 µg/m³) | MDB (WHO, 2013c), ICD-10 codes CVD: I00–I99; respiratory: J00–J99 | | APHENA study based on data from 32 European cities; coefficients adjusted for PM_10_ in two-pollutant model | Alternative to all-cause mortality analysis |
| O_3_, daily maximum 8-hour mean | | Mortality, CVDs and respiratory diseases, all ages | | A | CVD: 1.0049 (1.0013–1.0085); respiratory: 1.0029  (0.9989–1.0070) | >10 ppb (>20 µg/m³) | MDB (WHO, 2013c), ICD-10 codes CVD: I00–I99; respiratory: J00–J99 | | APHENA study based on data from 32 European cities; coefficients adjusted for PM_10_ in two-pollutant model | Alternative to the cause-specific assessment for O_3_ >35 ppb only |
| O_3_, daily maximum 8-hour mean | Hospital admissions, CVDs (excluding stroke) and respiratory diseases, age 65+ years | | A* | | CVD: 1.0089 (1.0050–1.0127); respiratory: 1.0044 (1.0007–1.0083) | >35 ppb (>70 µg/m³) | European hospital morbidity database (WHO, 2013f), ICD-9 codes CVD: 390–429; respiratory: 460–519 (ICD-10 codes I00–I52; J00–J99) | APHENA study based on data from eight European cities; coefficients adjusted for PM_10_ in two-pollutant model | | APHENA study based on all range of observed O_3_ concentrations, including levels <35 ppb; thus effects at O_3_ <35 ppb  are ignored |
| O_3_, daily maximum 8-hour mean | Hospital admissions, CVD (excluding stroke) and respiratory diseases, age 65+ years | | A | | CVD: 1.0089 (1.0050–1.0127); respiratory: 1.0044  (1.0007–1.0083) | >10 ppb (>20 µg/m³) | European hospital morbidity database (WHO, 2013f), ICD-9 codes CVD: 390–429; respiratory: 460–519 (ICD-10 codes I00–I52; J00–J99) | APHENA study based on data from eight European cities; coefficients adjusted for PM_10_ in two-pollutant model | | Alternative to the assessment for O_3_ >35 ppb only |
| O_3_, daily maximum 8-hour mean | MRADs, all ages | | B* | | 1.0154  (1.0060–1.0249) | >35 ppb (>70 µg/m³) | 7.8 days per year, based on Ostro and Rothschild (1989) | Ostro and Rothschild’s (1989) six separate analyses of annual data 1976–1981 of the United States National Health Interview Survey  Coefficient from single-pollutant model | | One study from the United States in 1989, used as a source of both RR and background rates |
| O_3_, daily maximum 8-hour mean | MRADs, all ages | | B | | 1.0154  (1.0060–1.0249) | >10 ppb (>20 µg/m³) | 7.8 days per year, based on Ostro and Rothschild (1989) | Ostro and Rothschild’s (1989) six separate analyses of annual data 1976–1981 of the United States National Health Interview Survey  Coefficient from single-pollutant model | | Alternative to the assessment for O_3_ >35 ppb only |

| NO_2_, long-term exposure | | | | | | | |
| --- | --- | --- | --- | --- | --- | --- | --- |
| Pollutant metric | Health outcome | Group | RR (95% CI) per 10 µg/m^3^ | Range of pollutant concentration to be quantified | Source of background health data | Source of CRF | Comments |
| NO_2_, annual mean | Mortality, all (natural) causes, age 30+ years | B* | 1.055  (1.031–1.080) | >20 µg/m³ | MDB (WHO, 2013c), rates for deaths from all natural causes (ICD-10 chapters I–XVIII, codes A–R) in each of the 53 WHO Regional Office for Europe countries, latest available data | Meta-analysis of all (11) cohort studies published before January 2013 by Hoek et al. (2013); RR based on single-pollutant models | Some of the long-term NO_2_ effects may overlap with effects from long-term PM_2.5_ (up to 33%); this is therefore recommended for quantification under Group B to avoid double counting in Group A analysis |
| NO_2_, annual mean | Prevalence of bronchitic symptoms in asthmatic children aged 5–14 years | B* | 1.021  (0.990–1.060) per 1 µg/m³ change in annual mean NO_2_ | All | Background rate of asthmatic children, “asthma ever”, in Lai et al. (2009) – western Europe: 15.8%, standard deviation (SD) 7.8%; northern and eastern Europe: 5.1%, SD 2.7%, with a recommended alternative of “severe wheeze” in Lai et al. (2009) – western Europe: 4.9%; northern and eastern Europe: 3.5%  Prevalence of bronchitic symptoms among asthmatic children  21.1% to 38.7% (Migliore et al., 2009; McConnell et al., 2003) | Southern California Children’s Health Study (McConnell et al., 2003); coefficient from two-pollutant model with OC (coefficients from models with PM_10_ or PM_2.5_ are higher) | Based on only one available longitudinal study providing NO_2_ coefficient adjusted for other pollutants  Supported by studies of long-term exposure to NO_2_ and lung function and by the wider evidence on NO_2_ and respiratory outcomes from other types of studies |

| NO_2_, short-term exposure | | | | | | | |
| --- | --- | --- | --- | --- | --- | --- | --- |
| Pollutant metric | Health outcome | Group | RR (95% CI) per 10 µg/m^3^ | Range of pollutant concentration to be quantified | Source of background health data | Source of CRF | Comments |
| NO_2_, daily maximum 1-hour mean | Mortality, all (natural) causes, all ages | A* | 1.0027  (1.0016–1.0038) | All | MDB (WHO, 2013c), rates for deaths from all natural causes (ICD-10 chapters I–XVIII, codes A–R) in each of the 53 countries of the WHO European Region, latest available data | APHEA-2 project with data from 30 European cities; RR adjusted for PM_10_ |  |
| NO_2_, daily maximum 1-hour mean | Hospital admissions, respiratory diseases, all ages | A | 1.0015  (0.9992–1.0038) | All | European hospital morbidity database (WHO, 2013f), ICD-9 codes 460–519; ICD-10 codes J00–J99 | APED meta-analysis of four studies published before 2006; coefficient from single-pollutant model  WHO (2013a) noted that the estimates for this pollutant–outcome pair were robust to adjustment to co-pollutants | Alternative to the estimates based on 24-hour NO_2_ average (preferred due to availability of more studies) |
| NO_2_, 24-hour mean | Hospital admissions, respiratory diseases, all ages | A* | 1.0180  (1.0115–1.0245) | All | European hospital morbidity database (WHO, 2013f), ICD-9 codes 460–519; ICD-10 codes J00–J99 | APED meta-analysis of 15 studies published before 2006; coefficient from single-pollutant model  WHO (2013a) noted that the estimates for this pollutant–outcome pair were robust to adjustment to co-pollutants |  |

Table notes:

Group A: Pollutant-outcome pairs contributing to the limited set of effects but considered already accounted for by summing those with an asterisk.

Group A*: Pollutant-outcome pairs contributing to the total limited set of effects (the effects are additive).

Group B: Pollutant-outcome pairs contributing to the extended set of effects but considered already accounted for by summing those with an asterisk.

Group B*: Pollutant-outcome pairs contributing to the total extended set of effects (the effects are additive).

Group B**: Only residual RADs to be added to total effect, after days in hospital, work days lost and days with symptoms are accounted for.

References

Hoek G, Pattenden S, Willers S, Antova T, Fabianova E, Braun-Fahrländer C, Forastiere F et al. (2012) PM10, and children’s respiratory symptoms and lung function in the PATY study. Eur Respir J 40, no. 3: 538-547.

Hoek G, Krishnan RM, Beelen R, Peters A, Ostro B, Brunekreef B, Kaufman JD (2013) Long-term air pollution exposure and cardio-respiratory mortality: a review. Environ Health 12, no. 1: 43.

IHME (2013). Global Burden of Disease Study 2010 (GBD 2010) – ambient air pollution risk model 1990–2010 [web site]. Seattle, WA, Institute for Health Metrics and Evaluation. <http://ghdx.healthmetricsandevaluation.org/record/global-burden-disease-study-2010-gbd-2010-ambient-air-pollution-risk-model-1990-2010>. Accessed 1 November 2013.

Jerrett M, Burnett RT, Pope III CA, Ito K, Thurston G, Krewski D, Shi Y, Calle E, Thun M (2009) Long-term Ozone Exposure and Mortality. The New Engl J Med 360 (11) (March 12): 1085–1095. doi:[10.1056/NEJMoa0803894](http://dx.doi.org/10.1056/NEJMoa0803894).

McConnell R, Berhane K, Gilliland F, Molitor J, Thomas D, Lurmann F, Avol E, Gauderman WJ, Peters JM (2003) Prospective Study of Air Pollution and Bronchitic Symptoms in Children with Asthma. Am J of Resp and Crit Care Med 168, no. 7 (October 1): 790–797. doi:[10.1164/rccm.200304-466OC](http://dx.doi.org/10.1164/rccm.200304-466OC).

Migliore E, Berti G, Galassi C, Pearce N, Forastiere F, Calabrese R, Armenio L et al. (2009) Respiratory symptoms in children living near busy roads and their relationship to vehicular traffic: results of an Italian multicenter study (SIDRIA 2). Environ Health 8, no. 1: 27.

Lai CKW, Beasley R, Crane J, Foliaki S, Shah J, Weiland S (2009) Global variation in the prevalence and severity of asthma symptoms: phase three of the International Study of Asthma and Allergies in Childhood (ISAAC). Thorax 64, no. 6: 476-483.

Ostro BD (1987) Air pollution and morbidity revisited: a specification test. J Environ Econ and Manag 14, no. 1: 87-98.

Ostro BD, Rothschild S (1989) Air pollution and acute respiratory morbidity: an observational study of multiple pollutants. Environ Res 50, no. 2: 238-247.

Weinmayr G, Romeo E, De Sario M, Weiland SK, Forastiere F (2010) Short-term Effects of PM10 and NO2 on Respiratory Health Among Children with Asthma or Asthma-like Symptoms: a Systematic Review and Meta-analysis. Environ Health Perspect 118, no. 4 (April): 449–457. doi:[10.1289/ehp.0900844](http://dx.doi.org/10.1289/ehp.0900844).

WHO Regional Office for Europe (2013b). Health risks of air pollution in Europe – HRAPIE project: Recommendations for concentration-response functions for cost–benefit analysis of particulate matter, ozone and nitrogen dioxide. Copenhagen, WHO Regional Office for Europe. <http://www.euro.who.int/en/health-topics/environment-and-health/air-quality/publications/2013/health-risks-of-air-pollution-in-europe-hrapie-project-recommendations-for-concentrationresponse-functions-for-costbenefit-analysis-of-particulate-matter,-ozone-and-nitrogen-dioxide>. Accessed 21 February 2014.

WHO Regional Office for Europe (2013c) European mortality database (HFA-MDB) [online database]. Copenhagen, WHO Regional Office for Europe. <http://data.euro.who.int/hfamdb/>. Accessed 14 November 2013.

WHO Regional Office for Europe (2013d) European detailed mortality database [online database]. Copenhagen, WHO Regional Office for Europe. <http://www.euro.who.int/en/data-and-evidence/databases/european-detailed-mortality-database-dmdb2>. Accessed 7 May 2014.

WHO Regional Office for Europe (2013e) European Health for All database (HFA-DB) [online database]. Copenhagen, WHO Regional Office for Europe. <http://data.euro.who.int/hfadb/>. Accessed 14 November 2013.

WHO Regional Office for Europe (2013f) European hospital morbidity database [website]. Copenhagen, WHO Regional Office for Europe. <http://www.euro.who.int/en/what-we-do/data-and-evidence/databases/european-hospital-morbidity-database-hmdb2>. Accessed 14 November 2013.

Woodruff TJ, Grillo J, Schoendorf KC (1997) The relationship between selected causes of postneonatal infant mortality and particulate air pollution in the United States. Environ Health Perspect 105, no. 6: 608.

Woodruff TJ, Darrow LA, Parker JD (2008) Air Pollution and Postneonatal Infant Mortality in the United States, 1999-2002. Environ Health Perspect 116, no. 1 (January): 110–115. doi:[10.1289/ehp.10370](http://dx.doi.org/10.1289/ehp.10370).
